# Supplementary figures and images for: Localization of oskar mRNA by agglomeration in ribonucleoprotein granules
Source: PLoS Genet. 2023 Aug 25;19(8):e1010877. doi: 10.1371/journal.pgen.1010877 (PMC10484445; doi:10.1371/journal.pgen.1010877)

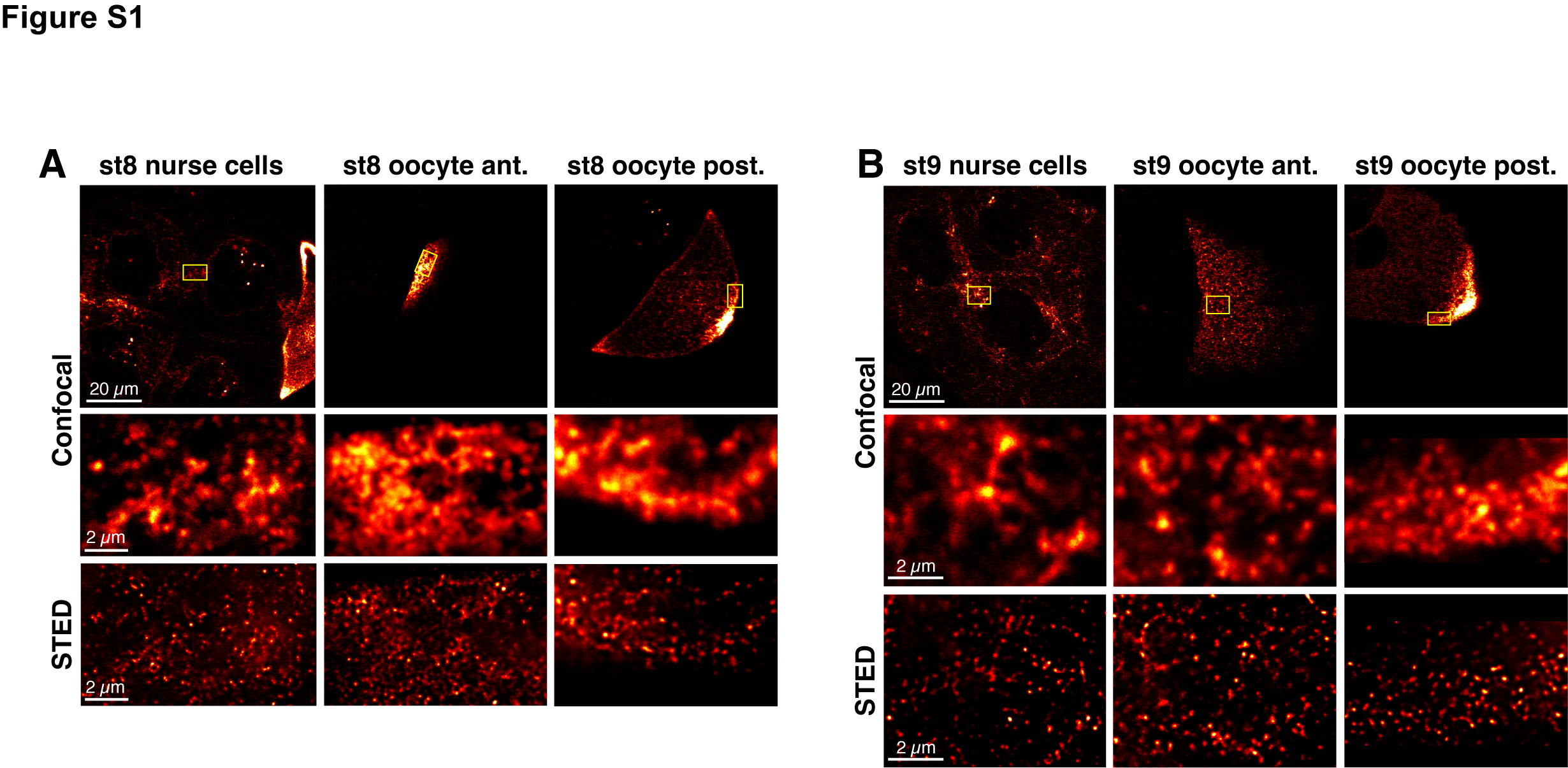

Supplement: S1 Fig — Images were taken of the nurse cells, the anterior region of the oocyte, and the posterior cortex of the oocyte in wild-type egg chambers at stage 8 (A) and stage 9 (B). osk mRNA was detected by smFISH. Panels in the top row are single confocal sections. The yellow boxes indicate ROIs imaged using STED microscopy as shown in the panels in the bottom rows. Panels in the middle rows show confocal images of each ROI prior to STED for comparison. Images are rendered using the Red Hot lookup table in Fiji and scale bars are indicated. (TIF) [file pgen.1010877.s002.tif]

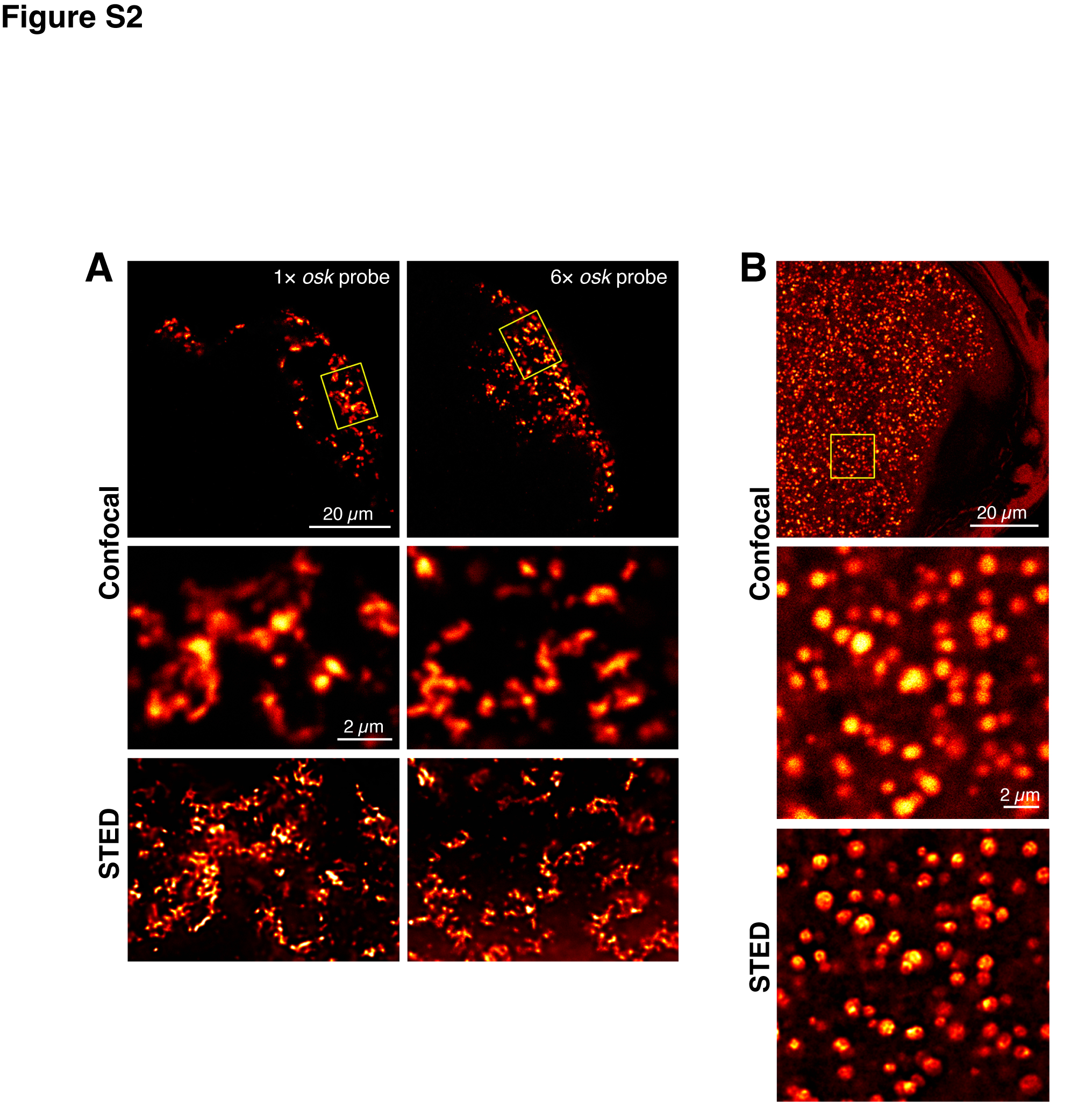

Supplement: S2 Fig — (A) Images of the posterior cortex of wild-type stage 13 oocytes. osk mRNA was detected by smFISH. Panels in the top row are single confocal sections. The yellow boxes indicate ROIs imaged using STED microscopy as shown in the panels in the bottom row. Panels in the middle row show confocal images of each ROI prior to STED for comparison. (B) Image of the posterior region of a stage 13 oocyte stained with Nile Red to detect lipid droplets. Confocal and STED images of the ROI indicated by the yellow box are shown in the middle and lower panels, respectively. Images are rendered using the Red Hot lookup table in Fiji and scale bars are indicated. (TIF) [file pgen.1010877.s003.tif]

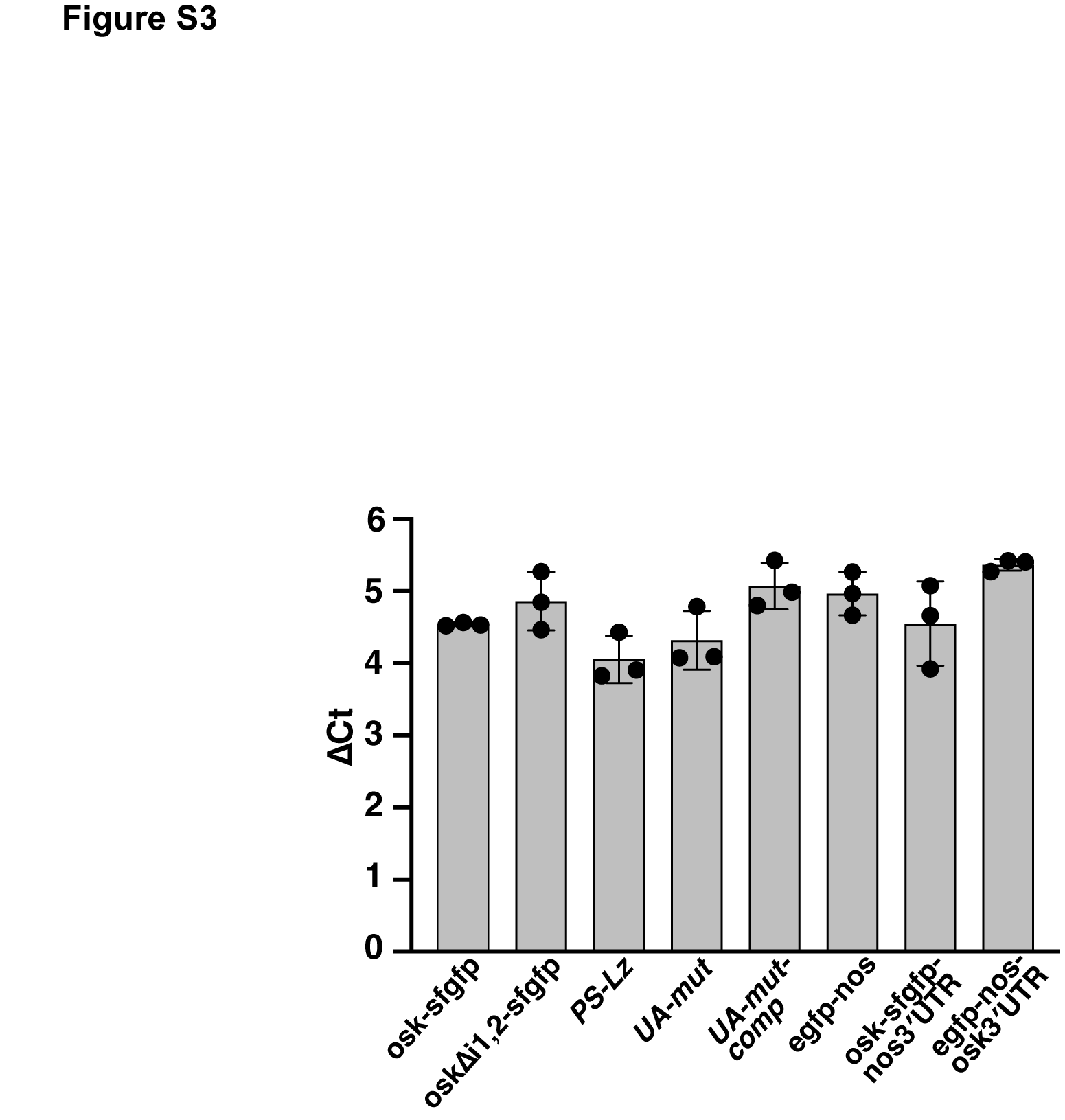

Supplement: S3 Fig — RT-qPCR quantification of transgenic mRNA extracted from early embryos, normalized to rpl7 mRNA. Individual data points and mean ± standard deviation are shown. Values are not significantly different from the osk-sfgfp control, as determined by one-way ANOVA and Dunnett’s multiple comparisons test. Source data for the graphs in S3 Fig are provided in S1 Data. (TIF) [file pgen.1010877.s004.tif]

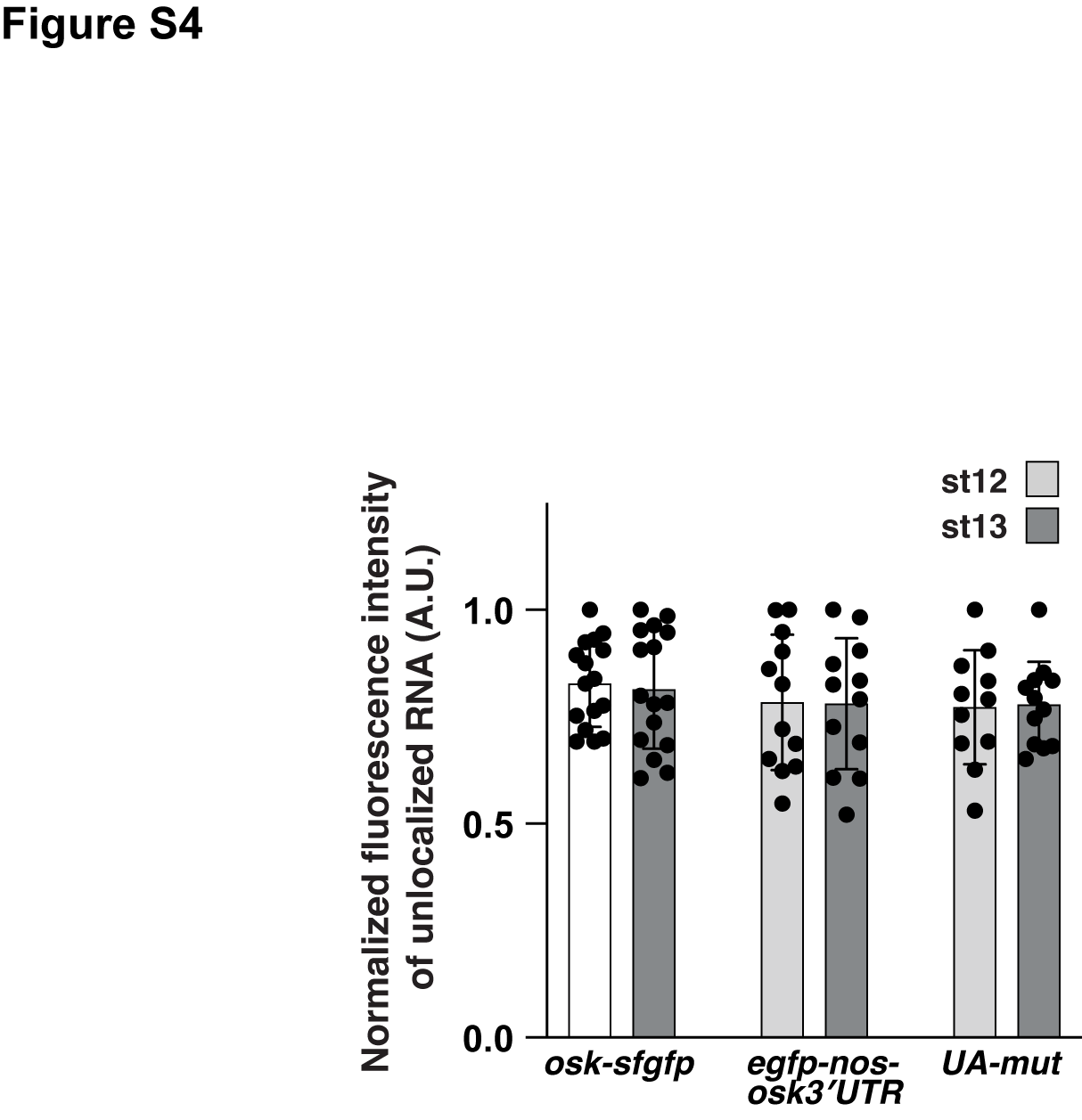

Supplement: S4 Fig — Fluorescence intensity measurements of unlocalized osk-sfgfp, egfp-nos-osk3′UTR and osk-sfgfpSOLEUA-mut mRNAs in stage 12 and stage 13 oocytes; n = 11–14 oocytes each. Individual data points and mean ± standard deviation are shown. The values are not statistically significant as determined by one-way ANOVA and Tukey’s post-hoc test. Source data for the graphs in S4 Fig are provided in S1 Data. (TIF) [file pgen.1010877.s005.tif]
