# Supplementary material for: Localization of oskar mRNA by agglomeration in ribonucleoprotein granules
Source: PLoS Genet. 2023 Aug 25;19(8):e1010877. doi: 10.1371/journal.pgen.1010877 (PMC10484445; doi:10.1371/journal.pgen.1010877)
Supplement: Attachment — Submitted filename: Response to Reviewers.docx [file pgen.1010877.s006.docx]

We are very pleased that the reviewers felt that our "experiments are well controlled, the images are convincing and the effects are quantified robustly" and found the work to be "important, original and timely" and we are grateful for their helpful suggestions. We have made most of the requested changes, which we believe have further strengthened the conclusions and made the manuscript more accessible, as elaborated below.

**Reviewer 1:**

We appreciate the point about the ability of RNAs to use different localization mechanisms depending on context and have added this to the manuscript, with acknowledgement.

I am supportive of publication but have a few minor suggestions for strengthening the manuscript.

1. The images in Figure 1 certainly seem to support an agglomeration process acting by stage 13. However, the conclusion would be more convincing if the authors could produce evidence that the punctate distribution of signals in the STED images is not an artefact of this imaging modality in the complex environment of the oocyte. For example, can the authors provide STED images of other structures (e.g. membrane compartments/nucleus) in late-stage oocytes that have a more uniform signal? And can the authors rule out that the punctate pattern stems from stochastic hybridization of probes within the founder granule (e.g., would the same signal be seen with a higher concentration of probe?).

We note that we had included images of germ granules (right-most panels in Figure 1), showing that they do not have the same appearance as founder granules. To provide additional evidence that the punctate distribution of the STED signals is not an artefact, we visualized lipid droplets, which are abundant particles similar in size to founder granules show that they have a uniform composition (new Figure S2). Lastly, we show that the pattern is the same over a 6-fold difference in probe concentration (new Figure S2).

2. The conclusion (made on multiple occasions) that agglomeration of oskar RNPs is independent of the EJC is, in this reviewer’s opinion, too strong based on the current data. They show that this process does not depend on the first two introns of oskar but it is formally possible that the EJC is required through a non-canonical mechanism. I recommend toning down this conclusion to state something along the lines of the EJC not appearing to be involved, at least not through the same process that occurs in mid-oogenesis.

We have toned down this conclusion in the abstract (lines 11-13), introduction (lines 147-148), results (lines 194, 202-203), and discussion (line 403).

3. In Figure 5C, does the K10 3’UTR enrich the RNA at the anterior of the oocyte? If this is the case, it may be that agglomeration is not occurring in this genotype because on an indirect effect of having too low a concentration of oskar RNA in the posterior region. I recognize that the conclusions are subsequently extended by the nos construct but nonetheless I recommend considering the addition of a caveat when introducing the K10 data.

We do detect some RNA at the anterior margin of the oocyte, although the majority is distributed over the cortex of the oocyte. The stage 10 oocyte we showed was likely in the process of nurse cell dumping, so exaggerated the effect. We have replaced panel A with a slightly younger stage 10 oocyte that is more representative. It was previously shown that the *osk* 3'UTR is required for localization through stage 10 (Kim-Ha et al., 1993), which we confirm in this experiment. The key question is whether it is required for the late phase and we show in panel B that it is not – note that the RNA is no longer enriched at the anterior margin of the late-stage oocyte, which is now visible on the left.

4. Figure 3 legend, panel J: should these read ‘at stages 10 and 13’ instead of ‘from 10 to 13’?

Corrected

5. Figure 7 legend: There is a typo in the line beginning ‘Transgenic RNAs were detected…’ (‘using with’).

Corrected

6. There is a figure legend for Figure S4 but it does not seem this figure is called out in the manuscript.

Apologies, we decided not to include this figure in the final manuscript as it didn't add new information and we neglected to remove the figure legend.

7. Figure 6. The authors may wish to consider color blind readers and change the colour scheme in panels B to I to green and magenta.

Very good point - we should have done this at the start. The color profile of the images is now green and magenta.

**Reviewer 2:**

Overall, the quality of the presented data is impressive and convincing. However, at least for a person not working on osk RNA, it is difficult to follow the experimental logic between the different blocks (see above) of the results. In my opinion, a graphical representation of osk RNA with its different elements and their positions and possible interaction(s) might really help. Secondly, it would be helpful to integrate the data into an RNA-centric osk model of how different signals might drive different phases of localization. As the authors mention the different contributions of dynein and kinesin, do they have data whether any of the motors contribute to the late accumulation? At least, it would be very informative to integrate motors into the working model.

We apologize for any confusion in the presentation. We agree that *osk* localization is a complex subject and we have made numerous textual changes, including simplifying the introduction and clarifying the logic, that we hope make the manuscript more accessible. Regarding the graphical representation of *osk* RNA, we now reference two reviews that provide schematics of the various components. We have not been able to do better than this ourselves. In the Discussion, we have now tried to clarify how different signals contribute to localization. Regarding motors, we and the Ephrussi lab have previously shown that the late *osk* accumulation depends on ooplasmic streaming, which is microtubule-dependent, but not on microtubule-dependent transport per se. We state on lines 111-113: "Notably, additional *osk* mRNA accumulates at the posterior during this later period, propelled by ooplasmic streaming rather than by kinesin-dependent transport (Glotzer et al., 1997; Sinsimer et al., 2011)". We have tried to clarify how different

Specific points for the authors:
• Figure 1: Though the authors are limited by 2D STED, it would be great to get an initial quantification of this data, e.g. changes in size, number of RNA molecules in agglomerates, or a quantification of clustering. As this is one of the core aspects of the paper, a quantification should be attempted in all figures where agglomerates are investigated (Fig. 4).

We appreciate the reviewer's interest in quantification but we are not comfortable reporting numbers that will be quite inaccurate. We can't accurately count the number of RNA molecules in agglomerates because we can't visualize the entire agglomerate in a single plane. Moreover, we use fluorescence intensity of transport RNPs (previously determined to contain 2-4 transcripts) as normalization and we can't accurately quantify fluorescence intensity in 2D images since the particles span at least 3 confocal sections in Z. We therefore rely on quantification of RNA number from confocal imagining where we can determine fluorescence intensities accurately (Figure 3K-M). We don't make any quantitative claims based on the STED imaging - we only describe the patterns that we observe.

• For purpose of clarity in line 200, the authors might indicate that both structure and sequence are required for late-phase accumulation. The current wording is contrived and unclear.

Thank you, we have made this change.

• In line 209, the text refers to a quantification in Fig. 4K,L, which is not part of the figure. It would seem the authors meant Fig.3. However, as mentioned above, a quantification of STED images would be encouraged, to clarify this point. The authors conclude, that SOLE mutants are unable to accumulate in late-stage founder granules. Would such a failure not increase the number of individual granules present, quantified in Fig. 3K? As mRNA levels remain constant, is the reporter mRNA localized to a different area?

Yes, we meant Fig. 3 and have made this correction. The failure to accumulate in founder granules would result in an increase in *osk* transport RNPs, containing 2-4 transcripts, throughout the oocyte. In Fig. 3K, we quantify only the localized particles (i.e., founder granules), which we define as those containing more than 4 *osk* transcripts (see Methods). We have not quantified the total number of unlocalized particles (note that only ~18% of *osk* RNA is localized at the posterior; Bergsten et al. 1999).

• The reasoning in line 252 is unclear, could the authors specify. How was Stau2 488 signal separated from GFP? Was GFP/488 signal observed in cycB granules? If osk-GFP or Stau2-488 was unspecific in germ granules, how can they be specific in founder granules? Please provide a clearer explanation. It is a nice idea to combine these aspects in the same embryos. However, perhaps it would have made more sense to perform this analysis in different embryos to ensure spectral separation.

We had explained that "Because the transgenes produce GFP-labeled proteins, to eliminate any contribution from residual GFP fluorescence that overlaps with germ granules and founder granules, we monitored founder granule association as far-red fluorescence signal colocalized with 488 fluorescence signal (Stau) that was not associated with 565 fluorescence (*CycB)*, which should represent Stau only." We apologize if this wasn't clear. We have rewritten this explanation: "Because the transgenes produce GFP-labeled proteins, to eliminate any contribution from residual GFP fluorescence we identified founder granules by the criteria they contain RNA (far-red) and Stau (488) but not *CycB* (565)."

• Please provide magnified insets for the images in all figures, as already done for the STED figures, to clearly show the granules.

We have added magnified insets to Figure 6 so that the colocalization or lack thereof is clear. The other figures simply show the differences in total accumulation (fluorescencent intensity) so we don't think it is helpful to complicate them with enlargments.

• Overall, do not use too many distinct terms for granules, which might be somewhat confusing for the general audience, e.g. RNPs, granules, bodies, etc.

We have restricted our wording to RNPs and granules, with explanations of what we mean	by each.
